# Is the pharmacy profession innovative enough?: meeting the needs of Australian residents with chronic conditions and their carers using the nominal group technique

**DOI:** 10.1186/1472-6963-14-476

**Published:** 2014-10-04

**Authors:** Sara S McMillan, Adem Sav, Fiona Kelly, Michelle A King, Jennifer A Whitty, Amanda J Wheeler

**Affiliations:** School of Human Services and Social Work, Population and Social Health Research Program, Griffith Health Institute, Griffith University, University Drive, Meadowbrook, QLD 4131 Australia; School of Pharmacy, Faculty of Medical and Health Sciences, University of Auckland, Auckland, New Zealand; School of Pharmacy, Griffith Health Institute, Griffith University, Gold Coast Campus, Parklands Drive, Southport, QLD 4215 Australia; School of Pharmacy, University of Queensland, Cornwall Street, Wooloongabba, QLD 4102 Australia; Griffith Health Institute and Centre for Applied Health Economics, School of Medicine, Griffith University, University Drive, Meadowbrook, QLD 4131 Australia

**Keywords:** Pharmacies, Nominal group technique, Prescribing, Innovation, Chronic disease, Australia

## Abstract

**Background:**

Community pharmacies are ideally located as a source of support for people with chronic conditions. Yet, we have limited insight into what innovative pharmacy services would support this consumer group to manage their condition/s. The aim of this study was to identify what innovations people with chronic conditions and their carers want from their ideal community pharmacy, and compare with what pharmacists and pharmacy support staff think consumers want.

**Methods:**

We elicited ideas using the nominal group technique. Participants included people with chronic conditions, unpaid carers, pharmacists and pharmacy support staff, in four regions of Australia. Themes were identified via thematic analysis using the constant comparison method.

**Results:**

Fifteen consumer/carer, four pharmacist and two pharmacy support staff groups were conducted. Two overarching themes were identified: extended scope of practice for the pharmacist and new or improved pharmacy services. The most innovative role for Australian pharmacists was medication continuance, within a limited time-frame. Consumers and carers wanted improved access to pharmacists, but this did not necessarily align with a faster or automated dispensing service. Other ideas included streamlined access to prescriptions via medication reminders, electronic prescriptions and a chronic illness card.

**Conclusions:**

This study provides further support for extending the pharmacist’s role in medication continuance, particularly as it represents the consumer’s voice. How this is done, or the methods used, needs to optimise patient safety. A range of innovative strategies were proposed and Australian community pharmacies should advocate for and implement innovative approaches to improve access and ensure continuity of care.

## Background

Similar to other countries, chronic conditions present an increasing burden in Australia [1]. As medication is generally needed to treat chronic conditions, pharmacists can provide further support in this area. Certainly, the Pharmaceutical Society of Australia’s (PSA) *‘Call to Action on Chronic Disease’* [2] comes at a most opportune time. The call is for pharmacists to expand their scope of services, thus realising their full potential to assist people with chronic conditions. While the provision of professional pharmacy services in Australia has increased, more needs to be done in preventative care, and to improve the health of people with a chronic condition [2].

Unpaid carers play an integral role in supporting people with chronic conditions. There are over 2.7 million carers in Australia, representing approximately 11.9% of the population [3]. This role could include managing the medication of the person they care for [4], hence carers are likely to interact with community pharmacy staff. Further insight is needed as to how community pharmacy can support carers, and subsequently, better assist the care receiver.

In order to be effective, healthcare services need to be of value to the end user; therefore, people with chronic conditions need to have more prominence in research [5]. This raises the question as to what pharmacy services do people want from community pharmacy to support them in managing their chronic condition/s. This is a particularly important question with respect to innovative pharmacy services, i.e. the introduction of a new service or approach in pharmacy [6]. In the context of this study, innovation was further defined to include the extension of the community pharmacist’s current role/s or services. Internationally, there have been significant changes to the pharmacist’s role and the services that community pharmacies can provide. This includes the provision of public health services [7], minor ailments schemes [8], pharmacist-administered vaccinations [9] and prescribing [10]. While there has been significant dialogue about extending the roles of Australian pharmacists [11], this has not eventuated into practice. There has been, however, research as to whether Australian residents actually want the types of services described above. Hoti *et al.* conducted one of two studies that explored the public’s view of pharmacist prescribing [12, 13], with the majority of participants accepting this expanded role within the community pharmacy setting. Although all study participants were regular pharmacy users and used at least one prescription medication, whether carers were involved and the proportion of participants living in a rural area was unclear [12]. This is important information, as people with chronic conditions are likely to have different experiences and needs to those who are carers. Differences would also be expected between urban and rural residents as healthcare access can differ depending on one’s location in Australia.

Furthermore, is innovation at the community pharmacy level driven by a clear understanding by pharmacy staff, i.e. pharmacists and pharmacy support staff, of what consumers/carers want? There is a need to explore this further; there is limited knowledge as to what community pharmacy staff believe people with chronic conditions and their carers want pharmacy to provide. If there are incongruent views between consumers, carers and pharmacy staff, differences will need to be explored further to ensure that pharmacy staff meet their consumers’ needs. This study aims to identify what innovations people with chronic conditions and their carers want from their ideal community pharmacy, and compare this with what pharmacy staff think consumers want community pharmacy to provide.

## Methods

### Study participants

Consumer participants with one or more chronic condition/s, or an unpaid carer for such a person, were purposively sampled [14], from four Australian regions; Mount Isa and Logan-Beaudesert (Queensland), Northern Rivers (New South Wales) and Perth (Western Australia). These areas vary in terms of accessibility to pharmacies, i.e. from being highly accessible in Perth, to modestly or remotely accessible in Mount Isa [15]. Participation included individuals from culturally and linguistically diverse populations (CALD) and Aboriginal or Torres Strait Islander peoples (IND).

The health professional groups consisted of either pharmacists or pharmacy support staff; pharmacy assistants were included as they are generally the first point of contact for consumers [16, 17]. It was preferable that pharmacists were currently employed, or had recent experience in the community pharmacy setting. This ensured that pharmacists had up to date knowledge of community pharmacy practice, and were therefore better placed to answer the research question. Recruitement for all participants involved snowball sampling [14] and dissemination of information to consumer health groups, professional organisations, and community pharmacies located within the study areas.

### Procedure

**Figure 1:**
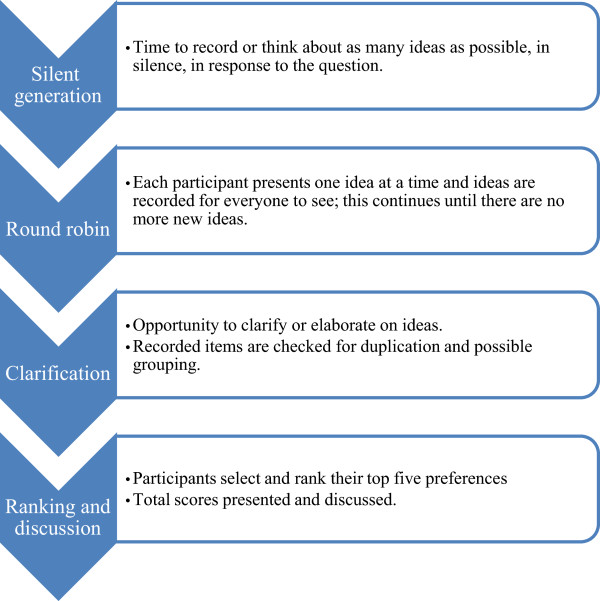
**Adapted version of Nominal Group Technique that is relevant to this study.**

The nominal group technique [18, 19], a highly structured process which facilitates the generation, discussion and ranking of participant ideas [20] (Figure 1), was used to elicit and compare opinions and priorities between consumers, carers and pharmacy staff [20, 21]. Participants were asked to *imagine their local pharmacy several years into the future: what services could they offer to help them to meet their individual health goals, or to best support them in their role as a carer?* To enable a direct comparison with the priorities of people with chronic conditions and their carers, pharmacy staff were directed to reflect on what they think their consumers would want, not what they would personally want.

Groups were conducted between December 2012 and April 2013, audio-recorded and transcribed verbatim. Three facilitators were deemed necessary to conduct the nominal groups effectively [22], and these roles were undertaken by three researchers of varying professional backgrounds, e.g. public health and pharmacy, as well as a consumer researcher. Informed consent was obtained from all participants prior to conducting the nominal groups. Griffith University Human Research Ethics Committee (PHM/12/11/HREC) provided study approval.

### Data analysis

A ranked list of priorities, i.e. ideas, generated from the ranking/discussion stage of each group (Figure 1) was reviewed to develop an initial analysis framework. This framework was discussed between four researchers who primarily facilitated the groups, with 23 themes condensed into 12 over-arching themes [22]. This final framework was then used to assist both qualitative and quantitative analysis, of which the latter process is detailed elsewhere [22]. All transcripts were analysed, via the framework, using the constant comparison method [23] and NVIVO.^©^ This paper reports the qualitative data relevant to *innovative services and roles*, one of the 12 themes identified.

## Results

Individuals participated in 15 consumer/carer groups (*n* = 103), two pharmacy support staff and four pharmacist groups (*n* = 35; Table 1). Participant numbers averaged six to seven per group (range, 2–14 people). Two over-arching themes were identified in relation to innovation; extended scope of practice for the pharmacist, i.e. new roles, and providing new or improved existing pharmacy services, i.e. increased access to the pharmacist and other healthcare professionals, medications, and information (Table 2). Additional quotes are provided in Table 3.

**Table 1:** **Participant data**

	Participants						
	Pharmacists	Pharmacy support staff	Consumers	Carers	Mixed consumer/Carer	Aboriginal or Torres Strait Islander peoples	CALD
Location	Groups						
Logan/Beaudesert (QLD)	1	1	2	1	2	1	1
Mt Isa (QLD)	1	0	1	1	1	1	0
Northern Rivers (NSW)	1	0	2	1	1	1	1
Perth (WA)	1	1	2^#^	0	1^*^	1	0
Total	4	2	7	3	5	4	2
*n*	22	13	54	17	32	35	17

n = total number of participants per group type.

^*^4 CALD participants participated in mixed consumer/carer group.

^#^2 CALD participants participated in one of the consumer groups.

**Table 2:** **Innovation themes generated from nominal group participants**

EXTENDED SCOPE OF PRACTICE FOR PHARMACISTS	• Repeat prescribing (Continuing supply of repeat medication)
	• Minor ailments scheme (e.g. for antibiotics, urinary tract infections)
	• Medication adjustments (e.g. dosing, side-effect concerns)
	• Point of care testing (e.g. blood cholesterol, glucose testing)
	• Pharmacist-administered vaccinations
NEW OR IMPROVED EXISTING PHARMACY SERVICES	*Increased access to pharmacist or other healthcare professionals*
	• New services: virtual pharmacy consultations, checking technicians, direct referrals to specialists from pharmacists, home visits/community health workers
	• Extended services: increased number of pharmacists in the pharmacy, forward pharmacy, Rolls Royce Service, co-location with other healthcare professionals/one-stop-shop
	*Increased access to medications and information*
	• New services: prompt dispensing and ordering, e.g. online and dispensing terminals, chronic illness card, drive thru service, service directory, case coordinator
	• Extended services: information sessions, health promotion, medication indication on prescriptions, prescription reminder service, home deliveries

**Table 3:** **Additional quotes illustrating themes**

**Extended scope of practice for pharmacists**	**Repeat prescribing (Continuing supply)**
	*Yeah, the pharmacist should be able to make prescriptions.* (Consumer_1208; Group_13)
	*…they* [pharmacists] *should be able to renew them but I think perhaps they should collaborate with the doctor…sometimes medication has changed…* (Carer_1149; Group_17)
	*Pharmacist needs to be able to prescribe medication, at least one that’s been already prescribed, ongoing medication, like contraception pill, or blood pressure medication.* (Pharmacy Assistant_2025; Group_11)
	*…pharmacists able to supply repeat therapies where appropriate.* (Pharmacist_2048; Group 18)
	*…why should I have to spend $60 going to see the doctor…every time I need a new script? It's daft.* (Consumer_1115; Group_8)
	*…I think the pharmacist profession is…much limited by legislation…if there is a side-effect for our medication…he* [pharmacist] *should be able to replace this medication for you.* (Consumer_1208; Group_13)
	*…they* [pharmacists] *can probably do that* [prescribe] *and free up maybe the system a little bit better…* (Pharmacy support staff_2042; Group_14)
	*Maybe a two month supply* [of medication]. *Because out here* [rural setting] *we have…a lot of compliance issues because people run out of their scripts…*(Pharmacist_2048; Group_18)
	**Medication adjustments**
	*Pharmacist reviews with the ability to adjust meds* [medication] (Pharmacist_2049; Group_18)
**New or improved existing pharmacy services**	**Increased access to pharmacist**
	*…they should have an extra chemist on the floor to explain all these things…* (IND_Consumer_1106; Group_4)
	**Co-location with other healthcare professionals and one-stop shop**
	*…a doctor within the pharmacy, so they come in, we can't do anything about it, straight in to the doctor”* (Pharmacist_2068) *“Taking that one step further, have an in-pharmacy setup for* [a] *virtual doctor…”* (Pharmacist_2062; Group_23)
	*…go from door to door and see all their health professionals in a one stop shop…* (Pharmacy Support Staff_2024; Group_11)
	*…have the pharmacy A type class, which is what we have now, and pharmacy B which is the specialist pharmacy who probably is in a health hub, and that's all they do. They don't sell sunglasses and they don't sell body stockings, they just dispense and they are highly trained and you probably pay a bit more…* (Carer_1217; Group_15)
	**Direct pharmacist referrals to health professionals**
	*…refer to professionals and get me into professionals in a timely manner…* (Consumer/Carer_1179; Group_Pilot2)
	**Community health workers**
	*…indigenous health workers out in the community, follow up the scripts…* (IND_Consumer_2; Group_3)
	**Prompt dispensing and ordering (online and dispensing terminals)**
	*…a USB script transmitter…some sort of system like a machine at the front or something that you could access, and bang, bang, bang, this is the script and it goes through to them* [pharmacy staff] *with a private password or key…it issues a script, and it will give you an estimated time when you can come back or it would be ready…* (Consumer/Carer_1041; Group_7)
	**Chronic Illness Card**
	*…a lot of the elders…travel a long way to go to funerals…That card would be priceless if they had that. So if they went a long distance, go to the chemist, zap it and do whatever they do and the information is right there for them. Because we all do travel…* (IND_Consumer_1158; Group_12)
	**Prescription Reminder Service**
	*…this is your last repeat, they* [pharmacies] *should give me a warning…* (CALD_Consumer_1231; Group_21)
	**Health promotion, information and service directory**
	*Information nights run by pharmacists and doctors for specific health conditions.* (Pharmacist_2053; Group_22)

### Extended scope of practice for pharmacists

Medication continuance was the most novel pharmacist role generated by all groups (consumer, carer, pharmacist and support staff), with an emphasis on renewing (repeat) medications, not diagnosing a new condition for a new medication. This role was expressed by some participants as being able to ‘make’, or ‘renew’ or ‘prescribe’ medication:

*Be able to prescribe medicine without a doctor's prescription…* (Consumer_1040; Group_6)

This was deemed useful for people taking medications regularly, at a consistent dosage, with the proviso of a limited time-frame, generally up to twelve months from the doctor’s initial prescription:

*…pharmacists should be able to renew prescriptions for people who are on the same medication year in, year out, for at least 12 months to take the pressure off doctors.* (Carer_1068; Group_17)

A reason for wanting pharmacists to have this extended role of medication continuance was to reduce the burden placed on GPs. Frustration about current practice was evident from consumer and carer groups, mostly from the inconvenience of making and attending GP appointments and their associated cost. Some non-urban participants described being charged a ‘prescription service’ to obtain ongoing (repeat) prescriptions when a GP was unavailable. Pharmacists were also frustrated with the administrative procedures associated when consumers ran out of their medication, and some consumers and carers did not understand why certain pharmacies provided an urgent supply of medication and others did not. This was particularly discussed by CALD participants; some did not understand why the pharmacist could not provide their medication when they had no prescription, particularly when the pharmacist was aware of their medication history. This resulted in additional stress:

*…I was very sick and then the chemist they said no way* [not supplying medication without a prescription]. *And sometime*[s] *you ring the doctor and they told you, next day, or one week…* (CALD_Consumer/Carer_1207; Group_13)

The types of medication suggested for continued supply were mostly those being used long term, such as blood pressure, insulin and cholesterol medication. Others, mostly pharmacy assistants, commented about the pharmacist being able to prescribe antibiotics or medication for simple ailments, thus alleviating the need to see the GP. This was viewed as a way to facilitate continuity of care for the consumer and reduce the burden on an already busy healthcare system. To enable this extended role, some consumers commented on having an agreed treatment plan with the GP. Carers particularly stressed the need for GP follow up or collaboration between GPs and pharmacists:

*…there can be a danger if they* [pharmacist] *just constantly renew it and you're not seeing your doctor.* (Carer_1013; Group_13)

One CALD consumer emphasised that pharmacists needed further ‘professional freedom’ to be able to do their job more effectively, i.e. to fully utilise their medication knowledge. This included the ability to change medication if there was a side effect, or to alter doses. The provision of *“point of care diagnostics”* (Pharmacist_2066; Group_23) was also mentioned by consumers and pharmacists, particularly for the monitoring of warfarin, blood glucose, cholesterol and blood pressure:

*…* [pharmacists] *should know who is a diabetic and who has blood pressure and have it automatically tested when they go to pick up their prescription…* (IND_Consumer_1106; Group_4)

### New or improved existing pharmacy services

#### Increased access to pharmacist or other healthcare professionals

Consumer and carer groups showed interest in pharmacy as part of a ‘one stop shop’, where the pharmacists *“work very closely in this health hub, or wherever they're situated, with the other* [healthcare] *providers…”* (Carer_1217; Group_15)*.* This carer also described having a more specialised, medical focused pharmacy in Australia, one that did not sell non-health related products. However, a pharmacist group thought their consumers would want a doctor located within a pharmacy, either physically or virtually. Consumer groups had a similar idea involving virtual access, but in relation to pharmacy staff. This involved something like Skype, and was seen as a way to improve privacy and reduce waiting time, allowing people who can’t, or don’t want to, physically enter the pharmacy to still access care:

*…rather than having to go and stand and telling my story in front of everybody else in the shop, I'd like to have a virtual one where you can have somebody talk to you on the line and you can just have a face-to-face.* (Consumer/Carer_1118; Group_13)

It was evident that consumers, carers and pharmacy support staff wanted increased access to other healthcare professionals in the pharmacy, such as nurse practitioners, physiotherapists and dieticians, to provide additional services:

…*instead of trying to train a pharmacist…you've got a nurse practitioner…and they've gone a little bit higher to get the betterment of qualification to give you the injection…* (Consumer/Carer_1214; Group_13)

Conversely, pharmacists diverged slightly from this view in relation to nurse-administered vaccinations, with discussion of the pharmacist conducting this role.

Home visits and utilising indigenous community health workers were discussed by an Aboriginal or Torres Strait Islander group. This involved monitoring a person’s healthcare and medication usage via community follow ups, having a yarn and getting to know the person, hence, assisting to ‘close the gap’, i.e. improve the health of Aboriginal or Torres Strait Islander peoples:

*If they see people aren’t coming to the chemist… they* [pharmacist] *should make it their business to go out to their house.* (IND_Consumer_1191; Group_3)

Increasing the opportunity to speak to a pharmacist was discussed in different ways via a consumer group, i.e. increasing pharmacist numbers in a pharmacy, a pharmacy assistant group, i.e. using checking technicians to relieve the pharmacist’s dispensing duties, and a pharmacist group, i.e. working predominantly outside the dispensary (forward dispensing) and offering a ‘Rolls Royce service’, at a price, to consumers:

*…you always get to talk to your pharmacist and your scripts are always ready for you so you jump the queue essentially…and you pay for that.* (Pharmacist_2000; Group_10)

Obtaining specialist referrals from the community pharmacist was also viewed as a way to improve healthcare access.

#### Increased access to medications and information

Improving healthcare access was discussed in relation to medications and information. Pharmacists considered consumers and carers would want dispensing or ‘script in’ machines, enabling more counselling time with the pharmacist and providing a faster service:

*This is quite controversial, but I think some people would want automatic dispensing…they'd have a computer, they'd type in their name and scan the prescription or something and it just dispenses to you.* (Pharmacist_2068; Group_23)

However, rapid dispensing was not discussed by the majority of consumers and carers. Only one participant wanted to be able to dispense their own medication, another wanted to fast-track the dispensing process with a system similar to photo processing booths. Instead, most consumers and carers wanted e-prescribing, or to be able to request their prescriptions online. This was emphasised as a way to reduce pharmacy queues and associated prescription paperwork, and help people with a physical disability to easily access their medication:

*…online prescriptions. So when the doctor types it out, why can't it…go straight to the chemist, and then hey you haven't got…papers to carry around…you can either go online* [to order it]…*or by phone…* (Consumer_1037; Group_8)

A chronic illness card was acknowledged as a tool to facilitate easier access to medication, without ‘the third degree’, i.e. being asked multiple questions by pharmacy staff:

*…a chronic illness card…so that it says you have a chronic condition…so that the pharmacist knows…* (Consumer/Carer_1206; Group_9)

This idea was also raised by an Aboriginal or Torres Strait Islander group, with participants describing what the card would incorporate, including their photo and medical history, known allergies, doctor/s details, and to be linked to preventative articles and information about their condition/s. These participants also thought it would be particularly useful for elders, especially when they travel or go ‘walkabout.’ Another participant wanted to have the medication indication listed on the prescription.

A prescription reminder system such as receiving an automatic alert when a new prescription was due or when their medication was running out, was another extended service suggestion:

*…before the due date…get an alert…Because a lot of elderly people forget…* (CALD_Consumer/Carer_1133; Group_7)

A pick-up and home delivery service was also mentioned by some consumers. However, a pharmacist group perceived that consumers would want an extended service of being able to obtain medication at any time, day or night, if needed. While consumer and carer groups did not request this much access, some did suggest a drive through service:

*You can just drive through with your ID and…here you go.* (Consumer/Carer_1118; Group_13)

Pharmacy support staff thought that consumers would want a simple service directory that provided information and contact details of healthcare and support organisations. However, having a case-coordinator to provide this information was discussed by consumers and carers:

*…accommodate my ideal case coordinator. Because the pharmacy isn't as intense as, say, even going to your GP or going to your specialist…house this person who would then have enough room to bring in guest speakers…have all the resources there, all the pamphlets we're needing and information…* (Consumer_1014; Group_15)

Extended services in the form of information evenings were also seen to be important:

*…the more interest and the more education you get into the pharmacies the better. Because a lot of people are using the pharmacist instead of their GP*. (Consumer_1116; Group_9)

## Discussion

This study strengthens support for Australian pharmacists to expand their scope of practice to include continued supply of repeat prescriptions. This was described in numerous ways by participants, with terminology including prescription renewal and prescribing. However, regardless of the terminology used, the end result was the same, i.e. that consumers and carers wanted to obtain a continual supply of the medication they regularly use, from their pharmacy, without having to go back to the doctor for a repeat prescription. Furthermore, pharmacist support staff and pharmacist groups also thought that consumers and carers would want this extended pharmacist role; they were correct in their assumptions.

Currently, when an Australian runs out of their prescription medication, a pharmacist can assist in the following ways by [24]:

(i) Contacting the prescriber and obtaining an ‘oral or faxed prescription.’ The prescriber sends the prescription to the pharmacist;
(ii) Providing three days (non-government subsidised) treatment, known as an ‘emergency supply.’ A follow-up prescription is not required; and
(iii) ‘Continued dispensing’ for one standard pack of statins and oral contraceptive pills only (e.g. 28 day supply). This is a relatively new arrangement in some Australian states and territories.

However, despite the above provisions, this study suggests that people with chronic conditions and carers are still having problems obtaining their prescription medication/s. This issue is linked with the inconsistencies in quantity and repeat prescription allowances for medicines for chronic conditions, i.e. 28 to 200 days’ supply, and zero to eleven repeats. Consequently, consumers can have difficulty synchronising their medications in terms of needing a new supply or prescription, particularly if they are taking numerous medications with different quantities and repeats. Furthermore, a pharmacist group described that, because of the limited GP appointments available in their rural area, consumers were being charged for a new (repeat) prescription without seeing their GP. Consumers and carers were frustrated with being unable to get a GP appointment in time, or being charged a fee just to pick up new prescriptions. While this study did not explore the experiences of our participants during their GP consultations, it is evident that consumers and carers wanted a more convenient system by involving the community pharmacist in prescription reminders and in the continual supply of their medication.

Groups also raised the issue of obtaining additional medication without a prescription when consumers had run out. While legislation permits the supply of up to three days’ medication or a single standard pack if the pack cannot be broken, e.g. cream, or eye drops, the supply of greater quantities without a prescription is illegal [25–27]. Our findings indicate that consumers and carers could not understand why larger quantities, e.g. their usual prescription, could not be supplied before the prescription had been received by the pharmacist, especially when the pharmacist was aware of their history and need for the medication. Ultimately, the prescription supply system could be made safer and more accessible to people by extending the continued dispensing legislation for pharmacists [28]. Pharmacists would then be freed from the conflict between acting within the law and acting in the patient's best interest. It would also align with Australia’s National Medicines Policy for more timely access to medicines [29], especially in rural and remote areas where time and travel are often barriers to obtaining healthcare [30]. Continued medication supply, if conducted appropriately, and collaboratively with GPs, could allow for more consumer contact with the pharmacist; something that participants wanted in this study.

This study cannot provide details with respect to ‘how’ pharmacists could provide a continued supply of medication to people with chronic conditions. However, an example of pharmacists working collaboratively with doctors to assist with medication continuance, and improve patient convenience, is demonstrated by the Pharmacy Anti-coagulation Management Services in New Zealand [31]. Pharmacists can monitor therapy, review results and provide dose changes under a GP standing order; prescribing rights are retained by the GP [32]. Healthcare policy makers and professional pharmacy bodies should consider two recent Australian reports documenting the movement towards ‘non-medical prescribing’ for pharmacists [33, 34], a move seen internationally with supplementary prescribing [35, 36]. Our findings align with aspects of supplementary prescribing, i.e. that a pharmacist prescribes in accordance to a clinical management plan, by continuing medication that has been approved by the patient’s medical prescriber [36].

There were differences in opinions between consumer, carer and health professional groups. Pharmacy staff overvalued the importance of rapid dispensing. Generally, consumer and carer groups did not want innovative dispensing machines; they wanted improved access to the pharmacist via new services, such as online, virtual pharmacy consultations and home visits. Although there are tele-health services in Australia, eligible healthcare providers require a Medicare provider number, which pharmacists currently cannot obtain [37]. Similar issues exist with home visits or follow-ups; pharmacists can only obtain re-imbursement if conducting a home medication review, or alternatively, charge the patient as a privately funded service. Participants also sought streamlined prescription access via extending services that some pharmacies may already provide (i.e. improved services), such as medication or prescription reminders, and electronic prescriptions, as well as the introduction of a chronic illness card containing their medical history. Consumers and carers also wanted easier access to a range of other healthcare professionals, with some commenting on having a ‘one-stop shop’, similar to a medical hub. However, this was described as more for the purposes of convenience [38]. Pharmacy assistants, not pharmacists, were more forthcoming with this idea.

### Strengths and limitations

This paper relies on self-reported data and does not present the quantitative (i.e. priority lists) results from the nominal groups. The majority of groups were conducted during working hours and may not be representative of people who work or have caring commitments in the day, e.g. parents. No specific information about the characteristics of the pharmacy that participants either used or worked in was sought. However, the nominal group method did allow people to talk about their experiences, giving participants an equal say in terms of responding to the question. Furthermore, this technique allows for the generation of more than one single idea, i.e. ideas are maximised and exhausted in each group. As the facilitators were not independent of the research, there was a risk of investigator-bias. However, this was done to facilitate the complex analysis process undertaken by the team [22]. Furthermore, the methodology used (i.e. nominal group technique) minimises the influences of the researcher [39], and a consumer researcher was involved to ground the findings in consumer experiences.

## Conclusion

Consumers and carers supported extending the role of the community pharmacist to include continued medication supply. Furthermore, this was one idea where views between the different groups, i.e. consumer, carer and pharmacy staff, were aligned. Continued medication supply could be via repeat prescribing or other management services, whichever method optimises patient safety. Pharmacists need to be aware that their consumers do not necessarily want a speedier, automated dispensing service, but seek greater access to them as a healthcare professional. Consequently, Australian community pharmacists and the pharmacy profession should consider these findings, given that they are what people with chronic conditions and carers want from their ideal pharmacy.

## References

[1] Australian Institute of Health and Welfare: **Australia's health 2012**. [http://www.aihw.gov.au/publication-detail/?id=10737422172&tab=3]

[2] Pharmaceutical Society of Australia: **Australians stay healthier: PSA's call to action on chronic disease**. [http://www.psa.org.au/download/submissions/call-to-action-on-chronic-disease.pdf]

[3] Australian Bureau of Statistics: **4430.0 - Disability, Ageing and Carers, Australia: Summary of Findings, 2012.** http://www.abs.gov.au/ausstats/abs@.nsf/mf/4430.0

[4] Carers Australia: **About carers**. [http://www.carersaustralia.com.au/about-carers/]

[5] Lempp H, Kingsley G (2007). Qualitative assessments. Best Pract Res Clin Rheumatol.

[6] Tann J, Blenkinsopp A: **Innovation in community pharmacy: accelerating the speed of change**. [http://www.natpact.info/uploads/innovation%20briefing%20-%20final.pdf]

[7] Agomo CO (2012). The role of community pharmacists in public health: a scoping review of the literature. J Pharm Health Serv Res.

[8] Pumtong S, Boardman HF, Anderson CW (2011). A multi-method evaluation of the Pharmacy First Minor Ailments scheme. Int J Clin Pharm.

[9] Grabenstein JD, Guess HA, Hartzema AG, Koch GG, Konrad TR (2001). Effect of vaccination by community pharmacists among adult prescription recipients. Med Care.

[10] Tonna AP, Stewart D, West B, McCaig D (2007). Pharmacist prescribing in the UK - a literature review of current practice and research. J Clin Pharm Ther.

[11] The Pharmacy Guild of Australia: **The roadmap: the strategic direction for community pharmacy.** http://www.guild.org.au/docs/default-source/public-documents/tab---the-guild/Strategic-Direction/here-.pdf?sfvrsn=0

[12] Hoti K, Hughes J, Sunderland B (2011). Pharmacy clients' attitudes to expanded pharmacist prescribing and the role of agency theory on involved stakeholders. Int J Pharm Pract.

[13] Bessell T, Mariott J, Emmerton L, Nissen L: **Improving Australians' access to prescription medicines: development of pharmacy practice models**. [http://www.guild.org.au/docs/default-source/public-documents/services-and-programs/research-and-development/Third-Agreement-R-and-D/2003-017/final-report.pdf?sfvrsn=0]

[14] Liamputtong P (2010). Research Methods in Health: Foundations for Evidence-Based Practice.

[15] Australian Population and Migration Research Centre: **Pharmacy access/remoteness index of Australia**. [http://www.adelaide.edu.au/apmrc/research/projects/pharia/pharia-info.html]

[16] Mott K, Eltridge F, Gilbert A, March G, Lawson T, Vitry A, Rao D, Weir D, Wade T, Anderson B (2005). Consumer experiences, needs and expectations of community pharmacy.

[17] Sheridan J, Kelly F, Basheer M, Jan R, Lee A (2011). Can I help you? A qualitative study of pharmacist and pharmacy assistant views on the role of pharmacy assistants in New Zealand. Int J Pharm Pract.

[18] Allen J, Dyas J, Jones M (2004). Building consensus in health care: a guide to using the nominal group technique. Br J Community Nurs.

[19] Harvey N, Holmes CA (2012). Nominal group technique: an effective method for obtaining group consensus. Int J Nurs Pract.

[20] Gallagher M, Hares T, Spencer J, Bradshaw C, Webb I (1993). The nominal group technique: a research tool for general practice?. Fam Pract.

[21] Drennan V, Walters K, Lenihan P, Cohen S, Myerson S, Iliffe S, Group SR (2007). Priorities in identifying unmet need in older people attending general practice: a nominal group technique study. Fam Pract.

[22] McMillan SS, Kelly F, Sav A, Kendall E, King MA, Whitty JA, Wheeler AJ (2013). Using the nominal group technique: How to analyse across multiple groups. Journal of Health Services Research and Policy.

[23] Glaser BG (1965). The constant comparative method of qualitative analysis. Soc Probl.

[24] Pharmaceutical Society of Australia: **Guidelines for the continued dispensing of eligible prescribed medicines by pharmacists**. [http://www.psa.org.au/download/guidelines/medication-management/continued-dispensing-practice-guide.pdf]

[25] Health (Drugs and Poisons) Regulation 1996. (QLD) ch 3 pt 4 div 2 s 194.

[26] Poisons Regulations 1965. (WA) pt 5 div 2 s 36.

[27] Poisons and Therapeutic Goods Regulation 2008. (NSW) pt 3 div 4 sub-div 2 s 45.

[28] National Health Act (2012). Commonwealth of Australia National Health (Continued Dispensing) Determination.

[29] Australian Government Department of Health and Ageing: **The national medicines policy document.** http://www.health.gov.au/internet/main/publishing.nsf/Content/national-medicines-policy

[30] Sav A, Kendall E, McMillan SS, Kelly F, Whitty JA, King MA, Wheeler AJ (2013). 'You say treatment, I say hard work': treatment burden among people with chronic illness and their carers in Australia. Health Soc Care Community.

[31] Shaw J, Harrison J, Harrison J: **Community pharmacist-led anticoagulation management service final report.** http://www.centraltas.co.nz/LinkClick.aspx?fileticket=B9rFT2EvcF8%3D&tabid=278&mid=1312 10.1111/ijpp.12097 24612135

[32] DHB Shared Services: **Community pharmacy warfarin service: community pharmacy anti-coagulation management (CPAM) service**. [http://www.centraltas.co.nz/LinkClick.aspx?fileticket=8zF_XLoZnIA%3D&tabid=278&mid=1048]

[33] Duckett S, Breadon P: **Access all areas: new solutions for GP shortages in rural Australia.** http://grattan.edu.au/report/access-all-areas-new-solutions-for-gp-shortages-in-rural-australia/

[34] Health Workforce Australia: **Health professionals prescribing pathway (HPPP) project - final report**. [http://www.hwa.gov.au/hppp]

[35] Pharmacy Council of New Zealand: **Pharmacist prescribers**. [http://www.pharmacycouncil.org.nz/cms_display.php?sn=232&st=1]

[36] Stewart DC, George J, Bond CM, Cunningham IT, Diack HL, McCaig DJ (2008). Exploring patients' perspectives of pharmacist supplementary prescribing in Scotland. Pharm World Sci.

[37] Australian Government Department of Human Services: **Telehealth for health professionals**. [http://www.medicareaustralia.gov.au/provider/incentives/telehealth/information-for-health-professionals.jsp]

[38] Wilson KA, Jesson JK, Staunton N (2000). Evaluation of a new health centre pharmacy: a case study. Int J Pharm Pract.

[39] Lloyd-Jones G, Fowell S, Bligh JG (1999). The use of the nominal group technique as an evaluative tool in medical undergraduate education. Med Educ.

[CR40] The pre-publication history for this paper can be accessed here: http://www.biomedcentral.com/1472-6963/14/476/prepub

